# Validation and clinical evaluation of a Polish translation of the Wisconsin Upper Respiratory Symptom Survey for Kids (WURSS-K)

**DOI:** 10.1186/s12955-021-01791-z

**Published:** 2021-05-24

**Authors:** Katarzyna Ostrzyżek-Przeździecka, Mariusz Panczyk, Aleksandra Ratajczak, Michał Bronikowski, Wojciech Feleszko

**Affiliations:** 1 Department of Pediatric Cardiology and General Pediatrics, Medical University of Warsaw, Zwirki i Wigury 63A, 02-091 Warsaw, Poland; 2 Department of Education and Research in Health Sciences, Medical University of Warsaw, Zwirki i Wigury 81, 02-091 Warsaw, Poland; 3 Department of Pediatric Pulmonology and Allergy, Medical University of Warsaw, ul. Zwirki i Wigury 63A, 02-091 Warsaw, Poland; 4 Department of Didactics of Physical Activity, Poznan University of Physical Education, Krolowej Jadwigi 27/39, 61-871 Poznan, Poland

**Keywords:** URTI, Respiratory infection, Wisconsin upper respiratory symptom survey, Children, Validity, Reliability

## Abstract

**Background:**

The Wisconsin Upper Respiratory Symptom Survey for Kids (WURSS-K) is a self-administered questionnaire developed to evaluate the severity of the common cold. It is a patient-oriented instrument that evaluates quality of life in an illness-specific manner to be used in children aged 10 years. The purpose of this study was to validate the Polish version of the Wisconsin Upper Respiratory Symptom Survey for Kids.

**Methods:**

The validation process consisted of five stages: forward translation, backward translation, cognitive debriefing, a pilot study (Study A and Study B), and statistical analysis. The first study (Study A, n = 10, aged 5–13) was conducted in the Emergency Room and an Outpatient Clinic of the Pediatric University Hospital in Warsaw. The purpose of the study was to obtain data for testing the convergent validity of the questionnaire. The second study (Study B, n = 56), consisted of children aged four to six enrolled in three kindergartens in the Warsaw suburbs. The obtained data were subjected to detailed statistical analysis.

**Results:**

The WURSS Kids Polish showed excellent reliability. The Cronbach’s alpha of the 13 items was 0.791 for the six symptom items and 0.854 for the seven functional items. The Jonckheere–Terpstra trend test was used to evaluate criterion validity. Compliance of the measurement performed independently by the examined person and the doctor on the first day was high (convergent validity). Each particular item was characterized by a different sensitivity to clinical change. The Guyatt’s Responsiveness index ranged from 0.083 to 0.464.

**Conclusion:**

The internal consistency of the measurements and cross-cultural adaptation of the Polish version of WURSS Kids was satisfactory. The WURSS Kids Polish is a reliable, valid, and responsive disease-specific questionnaire for assessing symptoms and QOL in Polish patients in the pediatric population with the common cold. It may be used both in clinical practice and for research among Polish children with URTI.

## Introduction

Upper respiratory tract infections (URTIs) are the most common cause of outpatient clinic visits among patients of all age groups [1, 2], accounting for more than 50% of pediatric medical consultations, and causing massive clinical and economic burden [3].

URTIs affect up to 25% of children under one year of age, and 18% of children one to four years of age [4, 5]. Preschool children are particularly prone to respiratory infections due to constant pathogen exposure among peers, different respiratory system anatomy, and immature immunity [6].

Because there are no specific biomarkers of the progression of URTIs, alterations in self-reported symptoms’ severity are considered an indicator of treatment effectiveness in various clinical studies. However, if changes in symptoms severity serves as a reliable indicator of the severity of illness in clinical studies, quantitative scales of such alterations should be established and validated [7–9]. At this time, an objective scale of URTIs’ symptoms may also serve as a differential diagnosis tool when suspecting a Severe Acute Respiratory Syndrome Coronavirus-2 (SARS-CoV-2) infection in the pediatric patient [10]. Since the symptoms in upper respiratory tract infections and COVID-19 vary, this scale might be a useful screening tool in the era of telemedicine and online consultations. Once the current pandemic is over, the tool can still be used in telehealth to provide more convenient, cost-effective care to patients [11].

The Wisconsin Upper Respiratory Symptom Survey (WURSS) is an evaluative illness-specific quality of life (QOL) instrument. It has been designed to assess the negative impact of acute upper respiratory infections, presumedly of viral etiology, including the common cold. Long (WURSS-44) and short (WURSS-21) versions of the questionnaire have been validated and compared with laboratory-based measures [12–14]. The WURSS has been widely used in numerous studies focusing on various respiratory diseases [15–20]. Recently, the validation of a WURSS questionnaire for kids (WURSS-K) was published [21]. It is the most up-to-date tool in the WURSS line of self-report instruments. It has also been considered a critical step in the research of URTIs in children as, up to this point in time, there have been no standardized tools to assess the course and severity of URTIs symptoms and their impact on the patients’ quality of life. A patient-oriented instrument that evaluates the patient’s QOL in an illness-specific manner provides new scientific research possibilities on large, multilingual populations. The standardized questionnaire can be used simultaneously in multiple healthcare centers worldwide; the results are comparable and thus considered equivalent. Being aware of the demand for such tools and the wide range of possibilities of its use, we aimed to translate the WURSS-K into the Polish language and, consequently, to validate the Polish version of the WURSS-K.

## Methods

WURSS-K is an instrument that measures illness-specific symptoms and their impact on the quality of life during upper respiratory tract infections (URTI). This tool includes 15 items: one global illness severity item (no. 1), six severity symptom-based items (no. 2–7), seven functional impact items (no. 8–14), and one comparison item for assessing change over the days of an illness (no. 15) (Table 1). Items from 1 to 14 are based on a 4-point ordinal Likert-type scale (0 = not sick/do not have this/not at all, 3 = very sick/very bad/very hard). The last item is based on a 5-point Likert-type scale. Happy and sad face representations are included along with the ordinal scales to facilitate survey completion by children.

**Table 1:** Content of the Wisconsin upper respiratory symptom survey for kids (WURSS-K)

Global severity items	Symptoms^a^	Functional impairments^b^	Global severity changes
1. How sick do you feel today?	2. Runny nose	8. Think	15. Compared to yesterday. I feel my cold is…
	3. Stuffy nose	9. Sleep	
	4. Sneezing	10. Breathe	
	5. Sore throat (hurts to swallow)	11. Talk	
	6. Cough	12. Walk, climb stairs, exercise	
	7. Feeling tired	13. Go to school	
		14. Play with friends	

^a^Directions for symptom-based items (2–7) ask respondents, “How bad are your cold symptoms (overall, since yesterday)”

^b^Directions for items on functional impairment (8–14) ask: “Since yesterday, how hard has it been to:”

### Translation

The First Step of preparing the Polish version of the WURSS kids’ questionnaire was forward translations done by two independent bilingual linguists. Subsequently, a translation panel consisting of linguists, pediatricians, and a statistician compared the two translations and collectively produced the final polish draft (WURSS kids—Polish v. 1.0).

The Second Step was a backward translation. Another bilingualist, not familiar with the WURSS-K questionnaire, translated the WURSS kids—Polish v. 1.0 from Polish into English. The translation panel gathered again and compared the original version of the WURSS-K and the back-translated versions for any inaccuracies. Particular attention was paid to ambiguous terms and the choice of vocabulary. The assumption was that the Polish version should be the most literal translation of the original version while maintaining apparent differences in the descriptions of symptoms such as “Runny nose” and “Stuffy nose.” After comparing all of the Polish and English versions prepared thus far, the second draft (WURSS kids—Polish v. 1.1) was composed.

**Fig. 1:**
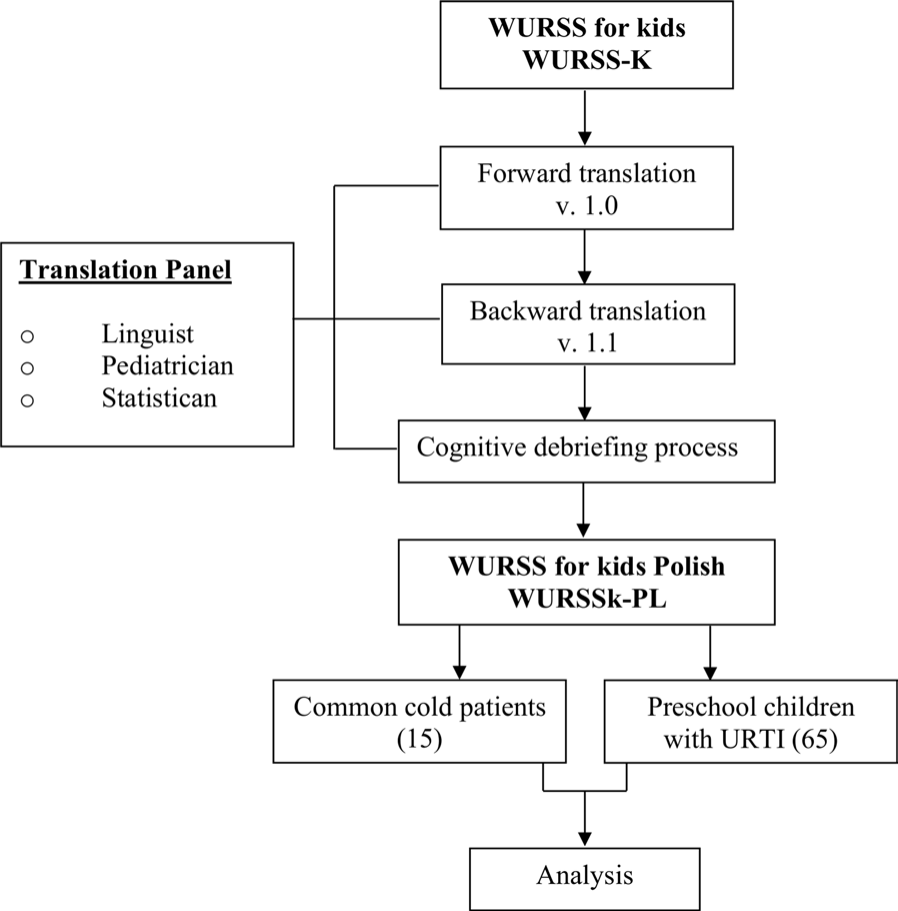
The process of translation and validation of the WURSS kids questionnaire in polish (WURSS—Wisconsin upper respiratory symptom survey, v—version)

The Third Step was cognitive briefing to test the comprehensibility of the WURSS kids—Polish. For this purpose, selected members of the translation panel interviewed 10 patients with URTIs (aged 5–7 years). The interview's purpose was to establish whether all words were understandable and unambiguous, and if the children could paraphrase or describe what they were asked, in their own words. The only question which needed a significant change in the translation was question 15: “*Compared to yesterday, I feel my cold is*…”. In situations where there was an absence of symptoms resulting from recuperation, four children responded, *“The same,”* which implied that s/he currently had a cold. Thus, the question was corrected while preparing the Polish version of the survey to ensure that we would receive a proper answer. The backwards translation of this sentence states: “*Compared to yesterday you are feeling*…”. After this change was made, the final version of the WURSS kids—Polish v. 1.2 (WURSSk-PL) questionnaire was created (Fig. 1).

### Pilot study

To obtain the data necessary for the validation process, we conducted two studies among children with URTIs symptoms, Study A and B.

#### Study design

The first study (Study A) was conducted in the Emergency Room and an Outpatient Clinic of the Pediatric University Hospital in Warsaw. Patients and parents were informed about the purpose of the study and its course. Participants who met the following criteria were included in the study: a “common cold” diagnosis with the onset of symptoms within 48 h. Exclusion criteria included allergic rhinitis, asthma, otitis, and any other chronic respiratory disease or acute lower respiratory tract infection. During the first visit, the participants and their guardians/parents were asked to complete the WURSSk-PL. At the same time, the questionnaire was filled in by the physician who was examining the patient. Participants were invited for a follow-up visit on the third day, and the same procedure was repeated in the presence of the same physician. All surveys were collected for comparison and to access convergent validity. The participants were allowed to take OTC medications of their choice during this study.

In the second study (Study B), 56 children aged four to six were enrolled. The children were recruited from three kindergartens in the Warsaw suburbs. At the beginning of the autumn/winter season, parents of all children in these classes received information about the possibility of participation in the study. Parents willing to enroll their children in the study received a qualification questionnaire that was developed specifically for the study. The study included children who met the inclusion and exclusion conditions and whose parents agreed to participate in the study. The parents of qualified children received a set of six questionnaires (one per day) and were asked to start filling in the questionnaires with their children starting on the second day after the presentation of symptoms of an upper respiratory tract infection (runny nose, cough, sneezing). The patient, assisted by a parent, completed the questionnaire daily from the second day for 6 days of the infection.

#### Ethics and consents

The Bioethical Commission of the Medical University of Warsaw approved this study (KB/212/2018). Initially, parents or legal guardians who verbally consented to the participation of their children in the study received participant information sheets containing all pertinent details regarding the aim and the course of the study, possible inconveniences related to participation, and information regarding the possibility of withdrawal at will. Parents signed an informed participation consent form prepared according to the Bioethical Commission's guidelines on the first day of the study. All personal information related to the study were encrypted by registration number and securely stored in a stand-alone, password-secured computer.

### Statistical analysis

To evaluate the psychometric properties of the WURSSk-PL, we assessed: internal consistency (Cronbach’s alpha), construct validity (confirmative factor analysis), criterion validity (Jonckheere’s trend test and Spearman’s rho correlation), and convergent validity (Kendall’s tau coefficient).

The collected data was examined for the inclusion of floor or ceiling effects. Adapted from Terwee et al. [22], it is accepted that floor or ceiling effects are not present if no more than 15% of respondents obtained the lowest or the highest possible score, respectively.

The analysis of the internal consistency of WURSSk-PL was based on the formula proposed by Cronbach [23]. An acceptable threshold for internal consistency for alpha-Cronbach greater than 0.70 was adopted [24]. Inter-item correlations were assessed, which were determined for each item within each of the distinct subscales. These correlations should not adopt values < 0.200.

Confirmatory factor analysis (CFA) was used to estimate the wellness of matching the obtained results to the imposed structure, the imposed structure of which results from theoretical assumptions developed for the original version of WURSS-K by Schmit et al. [21]. A two-factor structure (symptoms and function), excluding the first and last item, was expected. The expected values of indices recommended were as follows: χ^2^ divided by the degrees of freedom ≤ 3.00; the root mean squared error of approximation (RMSEA) < 0.08; the comparative fit index (CFI) and the Tucker–Lewis index (TLI) > 0.90 [25].

Criterion validity was estimated by assessing the correlation between the results of the WURSSk-PL self-assessment on a 4-point scale. For the assessment of correlation, Spearman’s rank correlation coefficient and Jonckheere’s trend test were calculated [26]. The use of the Jonckheere test allows for a null hypothesis: there is no correlation between the increase of self-assessment scores and the increase of the total scores. It is similar to the Kruskal–Wallis test in which the null hypothesis is that several independent samples come from the same population. However, with the Kruskal–Wallis test, there is no a priori ordering of the populations from which the samples are drawn. When there is an a priori ordering, the Jonckheere test has more statistical power than the Kruskal–Wallis test.

The magnitude of improvement of the respiratory symptoms was additionally assessed using the Guyatt's responsiveness index (GRI) [27]. This index corresponds with internal responsiveness and evaluates true changes that are assessed by external criteria. This index measures responsiveness by expressing an effect size for comparing the group of children who have improved with the group of children reporting no improvement. Guyatt’s Responsiveness Index for two groups (GRI = MID/√2MSE) is calculated as the ratio of the mean change in scores of children in the improving group (MID) divided by the SD (standard deviation) of the scores in the group of children reporting no improvement (√2MSE).

The Kendall's tau coefficient was calculated to estimate the convergent validity. This is a rank correlation coefficient used to measure the ordinal association between two measured quantities. The Kendall correlation between two variables will be high when observations have a similar rank between the two measurement: the results of the same instrument (WURSSk-PL) performed by the physician on Day 1 and Day 3 [28].

All statistical calculations were performed using the statistical package IBM® SPSS® Statistics, version 23, and Amos version 21. For all analyses, a *p*-level of < 0.05 was considered statistically significant.

## Results

### Participant characteristics

From among the 15 patients enrolled in Study A, 10 (5 girls and 5 boys, mean age M = 9.4; SD = 2.86, min–max = 5–13 years) attended their scheduled visits. Sixty-five patients were qualified for Study B, and 56 of them completed the 60-day follow-up (Table 2). The reasons for not completing the observation period include the absence of an upper respiratory tract infection during the study period and no compulsory follow-up visits at the end of the study.

**Table 2:** Characteristics of participants from Study B

Variable	Value
Age [years]	
M ± SD	5.2 ± 0.7
Range	4.0 – 7.0
Sex [no./total (%)]	
Female	26/56 (46.4)
Male	30/56 (53.6)
Height [M ± SD (cm)]	112.9 ± 4.94
Weight [M ± SD (kg)]	19.0 ± 3.2

M—mean, SD—standard deviation

### Item analysis

Item analysis concerned the participants' mean values from the first three measurements made with the WURSSk-PL. No SD was observed for any of the items. The results for most items were characterized by the lack of a normal distribution (critical ratio values for skewness and kurtosis outside of the range [− 2, 2]) (Table 3). There were no cases with the lowest possible result (no floor effect). Less than 5% of the cases achieved the highest possible result (no ceiling effect).

**Table 3:** Descriptive statistics for WURSSk-PL

Item	M	SD	Skew	CR	Kurtosis	CR
2	2.63	0.57	0.41	1.233	− 0.42	− 0.746
3	2.48	0.73	0.04	0.105	− 0.52	− 0.881
4	2.07	0.84	0.56	1.674	− 0.39	− 0.708
5	2.02	0.68	0.06	0.169	− 0.68	− 1.106
6	2.46	0.71	− 0.22	− 0.647	− 0.25	− 0.506
7	2.43	0.92	0.02	0.045	− 1.11	− 1.705
8	1.86	0.70	0.11	0.323	− 1.36	− 2.054
9	2.02	0.84	0.36	1.074	− 1.06	− 1.639
10	2.17	0.70	0.26	0.765	− 0.31	− 0.59
11	1.90	0.81	0.64	1.915	− 0.71	− 1.155
12	1.83	0.78	0.78	2.326	0.04	− 0.100
13	2.06	0.79	0.56	1.676	− 0.45	− 0.786
14	2.03	0.75	0.79	2.344	0.16	0.060

M—mean, SD—standard deviation, CR—critical ratio

### Internal consistency (Cronbach’s alpha)

As for internal reliability, the Cronbach’s alpha of the 13 items (based on the average of the scores for the first three days) was 0.791 for the symptom subscale and 0.854 for the functional subscale. There was no item-total correlation below 0.30 for any of the items, meaning that the subscales' internal consistency for both was high. Also for the entire scale (items from 2 to 14), internal consistency was very high (Cronbach's alpha was 0.902, average inter-item correlation 0.430, correlation item-total > 0.40). Items measuring global severity (“*How sick do you feel today?*”) and global change (“*Compared to yesterday, I feel my cold is*”) were not included in this test.

### Construct validity (confirmatory factor analysis)

**Fig. 2:**
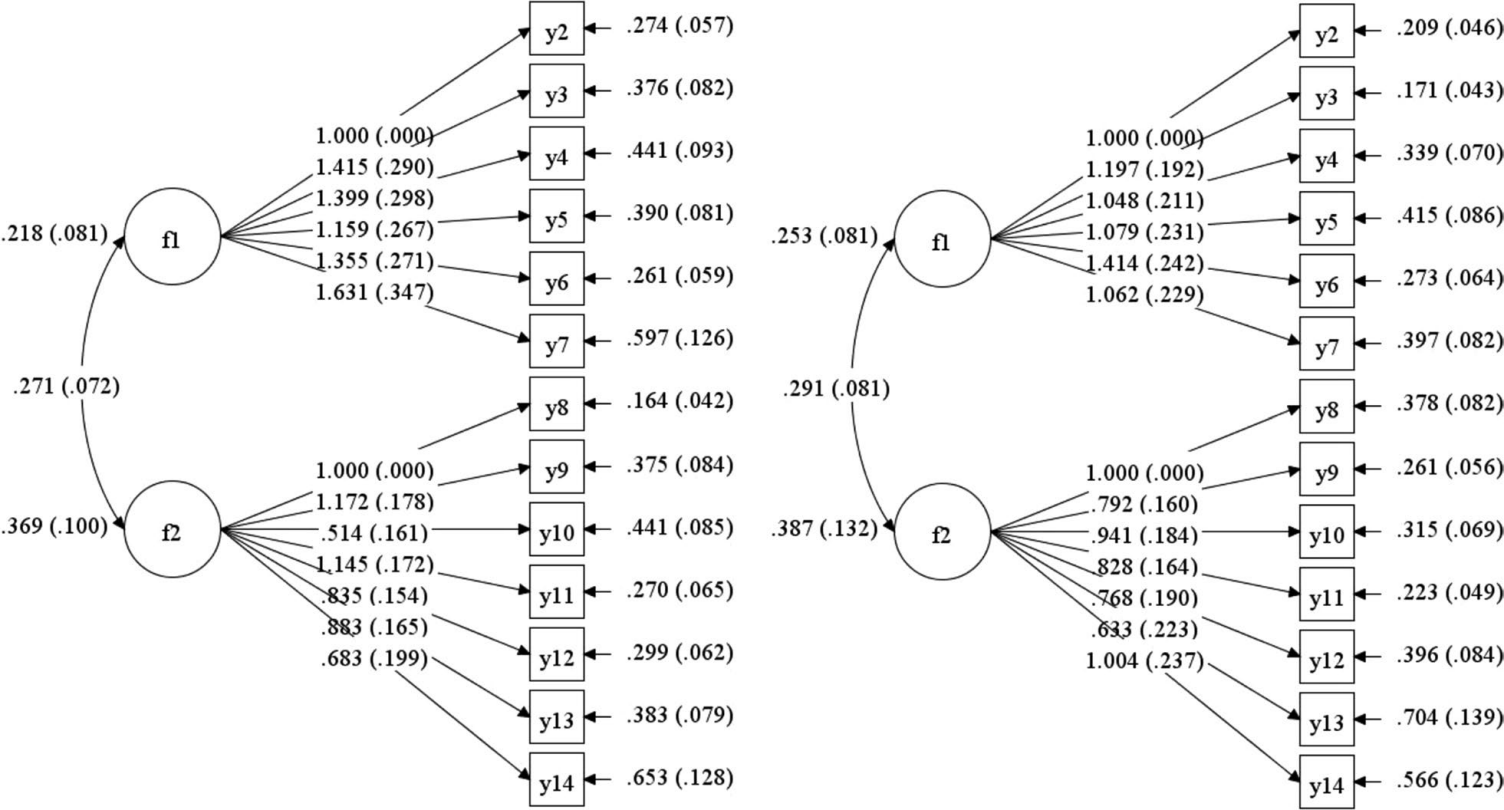
Structure of the Polish version of Wisconsin upper respiratory symptom survey for kids (the left diagram: the first day of measurement; the right diagram: the sixth day of measurement). Correlations between latent variables and items are represented with arrows. The number next to the items indicates how much variance was explained in the item. f1—symptom subscale, f2—functional subscale

A two-factorial solution (symptom and functional subscale) was in line with the original WURSS-K version's theoretical assumptions. As a result of the analysis, the ratio of the chi-square statistic to degrees of freedom (χ^2^/*df*) was found to be 2.46 (χ^2^ = 157.17, *df* = 64) for the first day and 3.27 (χ^2^ = 209.55, *df* = 64) for the sixth day of measurement, indicating a relatively good fit of the model. A χ^2^/*df* closer to 3.00 means that that model has a good fit. The RMSEA was 0.161 (90% CI [0.130–0.193]) and 0.202 (90% CI [0.172–0.232]), for the first and the sixth day of measurement, respectively, with the assumption that low RMSEA values (< 0.08) indicate a good fit of the model. The CFI was 0.774 and 0.678, and the TLI value was 0.724 and 0.607, respectively, indicating that this model does not have a very good fit. The details of the CFA are shown on the path diagram (Fig. 2).

### Criterion validity (Jonckheere’s trend test)

**Fig. 3:**
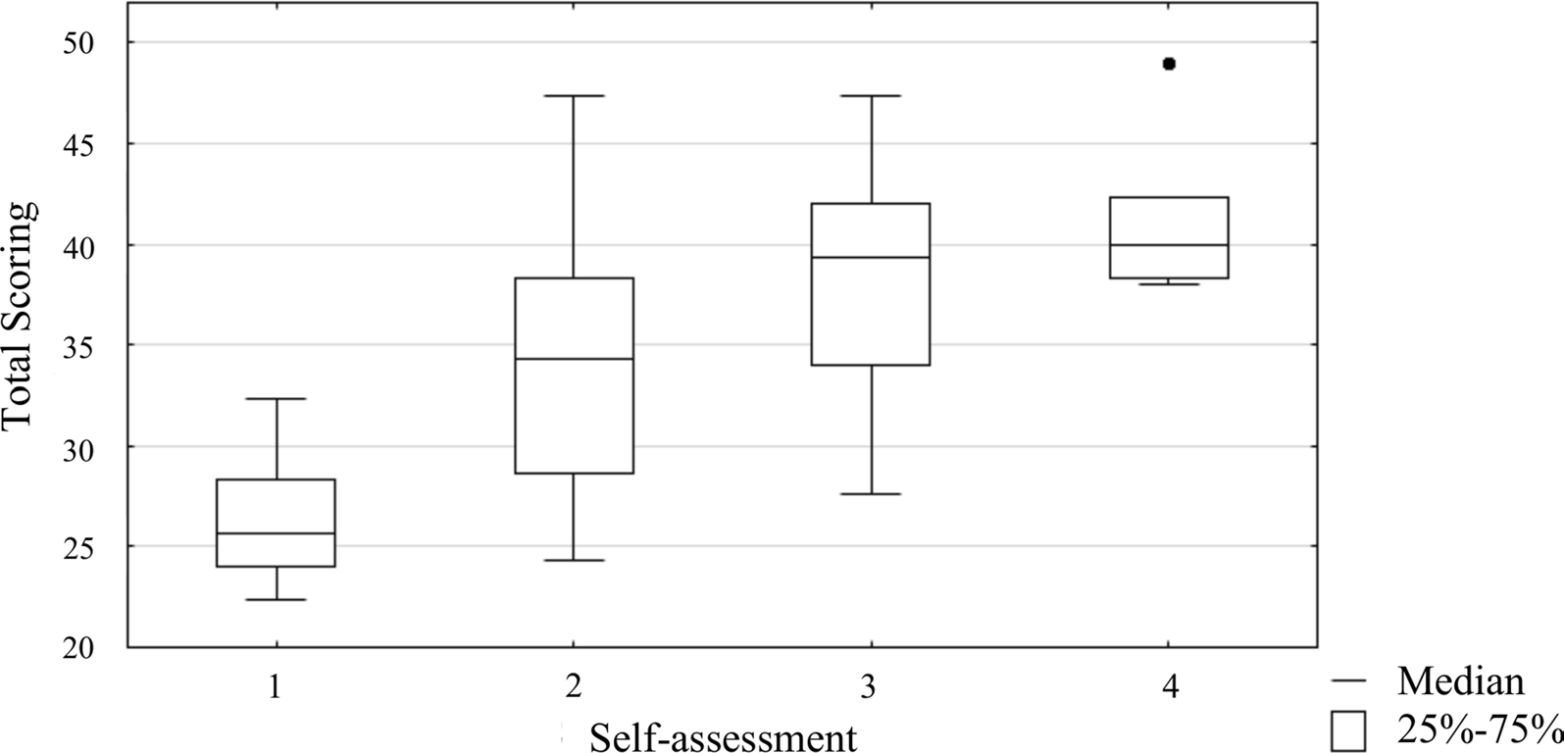
Criterion validity analysis

The total scores obtained for the answers to questions 2–14 were positively correlated with the self-assessment of the respondents in question one (Spearman’s ρ = 0.68, *P* < 0.001). The result of Jonckheere’s trend test (z = 5.183; *P* < 0.001) indicates that with the higher self-assessment of the subject, the cumulative score obtained from the WURSSk-PL measurement also increases (Fig. 3).

### Convergent validity (Kendall’s tau coefficient)

Convergent validity was evaluated by assessing the agreement of the WURSSk-PL measurement results obtained from the participants on Day 1 and Day 3 with the results of the same instrument performed by the physician on Day 1 and Day 3, respectively. Estimation of the agreement of the measurements was made by calculating the Kendall's tau coefficient. Convergence of the measurements performed independently by the examined person and the doctor on the first day was high (only two questions did not have any significant agreement). The results of the compliance assessment for Day 3 were slightly lower (Table 4).

**Table 4:** Ordinal association between two measured quantities

Item	Day 1^st^			Day 3^ed^		
	Kendall’s tau	Z	*P*-value	Kendall’s tau	Z	*P*-value
1	0.74	2.997	0.003	0.69	2.779	0.005
2	0.78	3.139	0.002	0.61	2.450	0.014
3	0.91	3.662	< 0.001	0.46	1.870	0.061
4	0.80	3.227	0.001	0.33	1.342	0.180
5	0.71	2.876	0.004	0.71	2.877	0.004
6	0.55	2.212	0.027	0.75	3.007	0.003
7	0.47	1.880	0.060	0.72	2.907	0.004
8	0.57	2.301	0.021	0.79	3.164	0.002
9	0.73	2.936	0.003	0.59	2.357	0.018
10	0.67	2.703	0.007	0.54	2.157	0.031
11	0.43	1.726	0.084	0.19	0.747	0.455
12	0.69	2.782	0.005	0.68	2.741	0.006
13	0.54	2.165	0.030	0.69	2.781	0.005
14	0.27	1.084	0.278	0.68	2.747	0.006

### Responsiveness test

The responsiveness of each item of the WURSSk-PL was checked by calculating the Guyatt’s responsiveness index. The responsiveness indices ranged from 0.083 to 0.464. The maximum index of 0.464 was obtained for the questions regarding “*Sneezing*” and question “*Since yesterday, how hard has it been to talk*”. The minimum index was obtained for the questions about “*Thinking*” and “*Feeling tired*” (Table 5).

**Table 5:** Responsiveness of each item

Item	GFI^a^
1. How sick do you feel today	0.169
2. Runny nose	0.307
3. Stuffy nose	0.332
4. Sneezing	0.460
5. Sore throat	0.258
6. Cough	0.348
7. Feeling tired	0.085
8. Think	0.083
9. Sleep	0.339
10. Breath	0.292
11. Talk	0.464
12. Walk, climb stairs, exercise	0.167
13. Go to school, preschool	0.283
14. Play with friends	0.292

^a^Guyatt’s responsiveness index

## Discussion

The results of the study show that the psychometric properties of the Polish version of WURSS for kids (WURSSk-PL) were satisfactory, and the use of this tool provides reliable and valid measurements. The validation process included the translation and psychometric analysis. Despite the satisfactory level of psychometric properties of internal consistency (Cronbach’s alpha), construct validity (confirmative factor analysis), criterion validity (Jonckheere’s trend test and Spearman’s rho correlation), convergent validity (Kendall’s tau coefficient), and responsiveness of each item of the WURSSk-PL, the two-factor structure has not been replicated.

The internal consistency of individual domains exceeded the recommended value (Cronbach's alpha for the entire scale was 0. 902) [23]. These results are similar to those obtained during validation of the original version of the WURSS-K [21]. The low level of random errors in our analysis that do not exceed 10% indicates excellent internal consistency reliability of the WURSSk-PL, which is a prerequisite for trusting the results of future measurements using this questionnaire.

Compared to the original version of the WURSS-K, the Polish version has shown similarities in construct validity. Both instruments have a two-factor structure (symptom and functional subscale). However, the factor analysis showed that the WURSSk-PL, unlike the WURSS-K, is more compatible with the established theoretical structure on the first day of measurements and is much worse on the sixth day. We assume that the discrepancy in describing symptoms, between parents and children in the United States and Poland is probably due to linguistic and cultural differences. This observation is confirmed by the significantly worse parameters for matching the collected data to the model structure being tested confirm this observation.

The analysis of criterion validity in our analysis was carried out following the concept that the self-assessment levels should be positively correlated with the global results obtained from the assessment using the WURSSk-PL. Even though the WURSS-K tool is considered to be both valid and reliable, this instrument has lacks clear criteria for applying each point on the Likert scale and all results obtained are based on the subjective observations of the patients’ and their respective physicians. However, Patient-Reported Outcome Measures (PROMs) are widely used and have been considered to be of great added value, as they may provide insight into the patients' perceived health and their needs, and enhance patient-professional communication and shared decision making [29].

Spearman's rank correlation coefficient and Jonckheere's test trend results confirm the above assumption, indicating a good criterion validity of the WURSSk-PL. Due to the lack of another available tools in the Polish language version an external criterion validity evaluation was not performed. Despite this limitation, an additional advantage of this validation is the convergent validity evaluation. Repeated measurements were performed with the same questionnaire in the same study group. Control measurements were performed by a doctor who acted in the role of a competent judge. The convergence of both measurements was calculated using the Kendall's coefficient of concordance, which ranges from 0 (no agreement) to 1 (complete agreement). The results confirm the excellent properties of the WURSSk-PL in terms of convergent validity, indicating that the questionnaire can be approved for use, and the results obtained in this way are largely in line with the doctor's observations during the patient's interview and physical examination. We want to emphasize that the questionnaire is a valuable asset that may be used widely in outpatient settings to assess the severity of symptoms. In the current times of the COVID-19 pandemics, it may also serve as a reliable screening questionnaire in telemedicine or for patients with URTI symptoms.

Another notable feature indicating the clinical usefulness of the WURSSk-PL in the assessment of the sensitivity of the questionnaire is its responsiveness towards clinical changes in the same patient over time (six consecutive days). We used the Guyatt's responsiveness index for this analysis and found that each particular item was characterized by a different sensitivity to clinical change. Among the most responsive are those elements of the questionnaire relating to such aspects of well-being as runny nose, stuffy nose, sneezing, cough, sleep, and speech. From a clinical point of view, we must admit that those elements are the most common symptoms of infection reported by children and their parents in the doctor’s office. A high response rate to those symptoms shows that the WURSSk-PL is a well-established questionnaire that focuses on the most common symptoms indicating the severity of the disease. On the other hand, “thinking” and “feeling tired” are the least responsive items. Both of these concepts are quite abstract for preschool children, resulting in a low clinical change.

The main limitation of this validation study is the sample size. At the level of about 500 participants, a larger sample size would allow us to perform an analysis using the model of the Item Response Theory (IRT), as it was done in the validation study of the original WURSS-K [21]. However, the use of the Classical Test Theory (CTT) has its advantages. It is a known and commonly used method, making it understandable for a larger group of recipients. The second limitation of this study is the lack of an assessment of the conformity of the measurement results made with the WURSSk-PL with the measurement results obtained by another tool, the so-called gold standard questionnaire, which the theoretical construct would be similar to the WURSSk-PL. However, such an analysis could not be carried out due to the lack of an existing Polish tool dedicated to assessing similar characteristics as the validated questionnaire.

The Polish version of the WURSS for kids meets all of the necessary criteria expected during a validation process and is recommended for clinical practice. Despite this, there are still some issues on which we need to focus our attention in further research. For instance, a larger study would allow us to perform more extensive psychometric analysis using an IRT model and compare the responsiveness of both the WURSS-K and WURSSk-PL and the correlation coefficient between the translated and original version at the same time.

## Conclusion

The Polish language version of the WURSS-K questionnaire is a valid, handy, and reliable instrument for evaluating children with upper respiratory tract infections. We conclude that this version of the questionnaire could be recommended for individual clinical assessment practice and observational studies in children with URTI in Poland.

## Data Availability

The datasets used and/or analyzed during the current study and Polish version of the WURSS-K are available from the corresponding author on reasonable request.

## Abbreviations

CFA: Confirmatory factor analysis
CFI: Comparative fit index
CTT: Classic test theory
GRI: Guyatt’s responsiveness index
IRT: Item response theory
OTC: Over the counter
QOL: Quality of life
PROMs: Patient-reported outcome measures
RMSEA: Root mean squared error of approximation
SARS-CoV-2: Severe acute respiratory syndrome coronavirus 2
SD: Standard deviation
TLI: Tucker–Lewis index
URTIs: Upper respiratory tract infections
WURSS: Wisconsin upper respiratory symptom survey
WURSS-K: Wisconsin upper respiratory symptom survey for kids
WURSSk-PL: Polish version of Wisconsin upper respiratory symptom survey for kids
